# *Gyrodactylus serrai* n. sp. (Gyrodactylidae), from the Near-Threatened Clanwilliam Sawfin, *Cheilobarbus serra* (Peters) (Cyprinidae, Smilogastrinae), in the Cape Fold Ecoregion, South Africa

**DOI:** 10.1007/s11230-024-10186-9

**Published:** 2024-10-15

**Authors:** Iva Přikrylová, Marliese Truter, Wilmien J. Luus-Powell, Albert Chakona, Nico J. Smit

**Affiliations:** 1 DSI-NRF SARChI Chair (Ecosystem Health), Department of Biodiversity, School of Molecular and Life Sciences, University of Limpopo, Sovenga, 0727 South Africa; 2 Water Research Group, Unit for Environmental Sciences and Management, North-West University, Potchefstroom, 2520 South Africa; 3 NRF-South African Institute for Aquatic Biodiversity (NRF-SAIAB), Makhanda, 6140 South Africa; 4 Department of Ichthyology and Fisheries Science, Rhodes University, Makhanda, 6140 South Africa

## Abstract

A new species of *Gyrodactylus* is described from the gills of the near-threatened Clanwilliam sawfin, *Cheilobarbus serra* (Smiliogastrinae) collected from the Matjies River, Cape Fold Ecoregion, Western Cape Province, South Africa. Morphometry and morphology of the haptoral hard parts (hamuli, bars and marginal hooks) of *Gyrodactylus serrai* **n. sp.** differ from the other known species of the genus in the smaller size of hamuli and the shape and size of marginal hooks. Furthermore, ITS rDNA for the new species is unique among available *Gyrodactylus* spp. data in GenBank. Based on the uncorrected *p*-distances, *G. serrai* **n. sp.** is genetically most closely related to *Gyrodactylus moroccensis* Rahmouni, 2023 and *Gyrodactylus pseudomoroccensis* Rahmouni, 2023 from two species of *Luciobarbus* (Barbinae) from northern Africa, with interspecific divergence of 8.7% and 8.8%, respectively. The presence of a median ridge in the terminal part of the ventral bar membrane at *G. serrai* **n. sp.** most probably represents a morphological link to the North African *Gyrodactylus* spp. that suggests a morphogenetic association across the African continent as a result of ancient waterways that facilitated the dispersion of cyprinids and their parasite fauna or an independent evolution event retaining similarities from a common ancestor. The description of *Gyrodactylus serrai* **n. sp.** represents only the second species of *Gyrodactylus* described from an endemic South African cyprinid host, underscoring the need for focused research on this group of fishes to provide a sound understanding of the parasitic communities of these highly threatened and poorly studied hosts.

## Introduction

The Cyprinidae is a highly diverse family of freshwater fishes with more than 1700 recognised species in 10 subfamilies and 158 genera (Fricke et al., 2023). Southern Africa boasts more than 80 cyprinid species of which 56 are known from the rivers of South Africa (Skelton, 2001). Recent taxonomic studies, however, indicate that the cyprinid diversity may be higher than currently thought (Skelton et al., 2018; Chakona et al., 2022). This is evident in South Africa’s Cape Fold Ecoregion (CFE) where there are currently 18 endemic cyprinid species, with an additional six candidate species awaiting formal taxonomic description (Skelton, 2001; Chakona et al., 2013, 2014, 2022; Skelton et al., 2018). Despite the growing discovery of higher cyprinoid diversity, limited attention has been given to studying the parasite communities associated with these fishes, in particular, those endemic to South African rivers (see Acosta et al., 2022). One example is the genus *Cheilobarbus* Smith which represents one of 33 genera of the subfamily Smiliogastrinae and contains only two valid species, the endangered *Cheilobarbus capensis* (Smith) and the near-threatened *Cheilobarbus serra* (Peters), both endemic to the CFE (Impson et al., 2017; Chakona et al., 2022). The subfamily Smiliogastrinae is a monophyletic clade of both diploid and tetraploid barbs consisting of 11 genera and 268 species endemic to Africa, and 19 genera with around 211 species distributed across Asia (Fricke et al. 2023). Within the Smiliogastrinae, the African representatives form a monophyletic lineage and is the sister group of the Asian genus *Systomus* McClelland (Yang et al., 2015; Ren and Mayden, 2016; Schedel et al., 2022). Both the African barbs and *Systomus* are nested among Asian representatives, making it likely that the Smiliogastrinae originated in Asia and dispersed into Africa in a “single” dispersal event with subsequent diversification on the continent (Yang et al., 2015; Ren and Mayden, 2016; Lavoué, 2020). Ren and Mayden (2016) estimated the crown age of the Smiliogastrinae as 37.4–31.2 Mya and the time of divergence between *Systomus* and the African barbs as 26.4 Mya (95% CI 28.4–20.5 Mya).

Monopisthocotylans of *Gyrodactylus* von Nordmann, 1832 are fish parasites with a global distribution parasitising a wide host range spanning many fish families (Harris et al., 2004; Paladini et al., 2011; Dos Santos et al., 2019; Lebedeva et al., 2021). Two decades ago, 409 valid species of the genus were confirmed (Harris et al., 2004) but the known diversity of the genus is thought to be over 500 species, with more than 80 *Gyrodactylus* spp. described in the last decade (PubMed search November 2023). Compared to the worldwide diversity of *Gyrodactylus*, the number of known African species (44) is still low, with only six species reported from cyprinid hosts distributed across the African continent [i.e., *G. ivindoensis* Price & Gery, 1968 (Gabon); *G. kyogae* Paperna, 1973 (Uganda); *G. nyingiae* Shigoley et al., 2023 (Morocco); *G. moroccensis* Rahmouni, 2023 (Morocco); *G. paludinosus* Truter et al., 2022 (South Africa); *G. pseudomoroccensis* Rahmouni, 2023 (Morocco)]. The Moroccan species, *G. nyingiae*, *G. moroccensis,* and *G. pseudomoroccensis*, are parasites of species of *Luciobarbus* Heckel (Barbinae) while the remaining three *Gyrodactylus* spp. infect various species of *Enteromius* Cope (Smiliogastrinae).

In comparison to the rest of the world, the gyrodactylid diversity in South Africa is rather low, with only eight recognised species of *Gyrodactylus*. Two of these species are known from marine hosts and six occur in freshwater environments with only two species reported from cyprinid hosts (Truter et al., 2022; Maduenyane et al., 2023). *Gyrodactylus eyipayipi* Vaughan et al., 2010 and *G. molweni* Christison et al., 2021 were found on marine fishes, *Sygnathus acus* Linnaeus and *Chelon richardsonii* (Smith) respectively, along the coast of the Western Cape Province (Vaughan et al., 2010; Christison et al., 2021). The freshwater species described from South Africa are *G. paludonisus* from *Enteromius paludinosus* (Peters), *G. transvaalensis* Prudhoe & Hussey, 1977 from *Clarias gariepinus* (Burchell) and *G. ulinganisus* García-Vásquez et al., 2011 from *Oreochromis mossambicus* (Peters) (Prudhoe and Hussey, 1977; García-Vásquez et al., 2011; Truter et al., 2022). Additionally, two freshwater species, *G. thlapi* Christison, Shinn & Van As, 2005 and *G. sturmbaueri* Vanhove et al., 2011 parasitise *Pseudocrenilabrus philander* Weber that were originally described from Botswana and Zambia, respectively, has also been reported from South Africa (Christison et al., 2005; Truter et al., 2016; Zahradníčková et al., 2016). The sixth species *G. sprostonae* Ling, 1962, which naturally infects *Carassius auratus* (Linnaeus) and *Cyprinus carpio* Linnaeus (invasive species in South Africa), underwent a spillover event and already parasitises a native endemic cyprinid species *Labeobarbus aeneus* (Burchell) (Maduenyane et al., 2023).

During recent surveys that aimed to document the parasite communities associated with endemic freshwater fishes of the CFE, undescribed *Gyrodactylus* specimens were collected from the Clanwilliam sawfin, *Cheilobarbus serra* (Truter et al., 2023). Previous studies indicated that integration of molecular data, in particular, the internal transcribed spacer (ITS) ribosomal DNA, and detailed morphological characterisation is the most rigorous approach for the delineation of species boundaries in the genus *Gyrodactylus* (Dos Santos et al., 2019; García-Vázques et al., 2019; Christison et al., 2021; Jin et al., 2024). In keeping with this recommendation, the present study describes a new species, *Gyrodactylus serrai* **n. sp.** from the endemic *C. serra* in South Africa, based on the combination of morphological and molecular (ITS rDNA) data. This is the first monopisthocotylan to be described from a host of the genus *Cheilobarbus*. The conservation implications of this discovery on a threatened narrow-range endemic species are also discussed.

## Materials and Methods

In December 2022, 15 individuals of Clanwilliam sawfin, *C. serra* [total length (cm) = 14–35 (23.2)] were collected from the Matjies River (32º30’00”S, 19º19’57”E), Cederberg Wilderness Area, Western Cape Province, South Africa as part of a monitoring survey by the local conservation agency (CapeNature) and biodiversity inventory of endemic species in South Africa. Ethical approval for the study and research permits for fish collection was obtained prior to sampling from the North-West University AnimCare Research Ethics Committees (NWU-00781-22-A5) and CapeNature (CN44-87-23462; CN16-87-23461) respectively. Fish were caught using fyke nets and transported to a temporary field station where they were kept in aerated water collected from the sampling site following the NWU-approved protocol (SOP NWU-00272-17-A5) for the temporary holding of fish. Each fish was humanely killed by percussive stunning and cervical transection and dissected following the protocol for the Ethical Handling of Ectothermic Vertebrates (SOP NWU-00267-17-A5). All external and internal organs were screened for the presence of parasites using a Nikon SMZ445 Zoom Stereo Microscope (Nikon, Tokyo, Japan). Monopisthocotylan parasites were preserved for both morphometric and molecular analysis as per Řehulková et al. (2018). The terminology and methods of measurements of the parasite’s body, male copulatory organ and haptoral hard parts followed Christison et al. (2005). Photomicrographs were captured using a compound microscope (Nikon Eclipse N*i*, Nikon, Tokyo, Japan) equipped with differential interference contrast, a computerised digital camera system and NIS-Elements BR 4.60© software for image analysis. All measurements are presented in micrometres, unless otherwise stated, and are presented as the range with the mean and number of measurements in parentheses. Drawings of the sclerotised structures were made with the aid of a camera lucida attached to a phase contrast compound microscope (Olympus BX51, Olympus, Tokyo Japan) at the Parasitology Lab, Department of Botany and Zoology, Faculty of Science, Masaryk University, Brno, Czech Republic. The line drawings were digitised using Adobe Illustrator© software (Adobe Inc., San Jose, CA, USA) and a Wacom Intuos Pro drawing tablet (Wacom, Saitama, Japan), following Truter et al. (2022).

Before depositing the specimens in museum collections, the specimens in Ammonium Picrate Glycerine (APG) were transferred into Canada balsam following Ergens (1969). Type and voucher specimens, as well as molecular voucher [=hologenophore (Pleijel et al., 2008)] were deposited in the parasitological collection in the National Museum, Bloemfontein, South Africa (NMB) and in the Helminthological Collection of the Institute of Parasitology, Biology Centre of the Academy of Sciences of the Czech Republic, in České Budějovice (IPBCAS).

Genomic DNA was isolated from the anterior part of the parasites’s bodies (*n* = 3) using the DNeasy Blood and Tissue Isolation kit (Qiagen, Hilden, Germany), according to the manufacturer’s protocol. DNA was eluted in 50 μl. The ITS1-5.8S-ITS2 region of the rDNA was amplified with the primers ITS-1F (5′-GTTTCCGTAGGTGAACCT-3′; Rokicka et al., 2007) and ITS-2R (5′-TCCTCCGCTTAGTGATA-3′; Matějusová et al., 2001). Polymerase chain reactions (PCR) had a final volume of 25 μl: 3 μl of DNA extraction supernatant, 10.5 μl DreamTaq PCR Master Mix (2×) (Thermo Fisher Scientific, Waltham, Massachusetts, USA), 9.5 μl of nuclease-free water, and 1 μl of each PCR primer. The temperature profile of the PCR followed Přikrylová et al. (2013), denaturation of 3 min at 95 ºC and 30× cycles of amplification (1 min at 94 ºC, 1 min at 50 ºC and 1 min 30 s at 72 ºC). PCR products were visualised on 1% agarose gel using GelRed® (Millipore Sigma, Burlington, Massachusetts, USA). PCR amplicons were purified and sequenced at Inqaba Biotechnical Industries (Pty) Ltd using PCR primers. Contiguous sequences were assembled using Geneious v. 7.1.3 (Kearse et al., 2012).

The newly generated sequence of ITS1-5.8S-ITS2 rDNA region was subjected to a BLAST search and sequences for *Gyrodactylus* spp. with an identity of 85% and above and a coverage of 75% and above were retrieved from the GenBank database and included in the final alignment. *Gyrodactylus alekosi* Přikrylová, Blažek and Vanhove, 2012a, 2012b and *Gyrodactylus rysavyi* Ergens, 1973 were selected as outgroups following Přikrylová et al. (2013). The list of sequences included in a phylogenetic reconstruction is provided in Table 1. Selected sequences were aligned with the aid of the Fast Fourier transform algorithm in MAFFT (Katoh et al., 2019) applying the L-INS-I method. The alignment was manually trimmed to unify the length and followed by trimming using trimAl v.1.2 (Capella-Gutierrez et al., 2009). The optimal phylogenetic model was selected based on the Akaike Information Criterion (AIC; Hurvich and Tsai, 1989) using ModelFinder (Kalyaanamoorthy et al., 2017) in IQ-TREE v2.2.

**Table 1:** List of *Gyrodactylus* spp., their host species, country of collection, and GenBank accession number for ITS rDNA sequences used for phylogenetic reconstruction

*Gyrodactylus* species	Host species (Family)	Country of collection	Accession Number	Reference
*Gyrodactylus alekosi*	*Clarias gariepinus* (Clariidae)	Mozambique	FR850682	Přikrylová et al. (2012b)
*Gyrodactylus banmae*	*Danio renio* (Cyprinidae)	China	MW353802	Jin et al. (2022)
*Gyrodactylus gibraltarensis*	*Luciobarbus graellsii* (Cyprinidae)	Spain	OR773480	Rahmouni et al. (2023)
*Gyrodactylus gymnodiptychi*	*Gymnodiptychus dybowskii* (Cyprinidae)	China	MH445967	Zhang et al. (2023)
*Gyrodactylus jiroveci*	*Barbatula barbatula* (Nemacheilidae)	Czech Republic	AM502860	Přikrylová et al. (2008)
*Gyrodactylus kobayashii*	*Carassius auratus* (Cyprinidae)	China	KJ755085	Unpublished
*Gyrodactylus mhaiseni*	*Alburnus sellal* (Leuciscidae)	Iraq	OR773082	Benovics et al. (2024)
*Gyrodactylus monogolicus*	*Oreoleuciscus potanini* (Leuciscidae)	Mongolia	OQ913866	Lebedeva et al. (2023)
*Gyrodactylus moroccensis*	*Luciobabrbus rabatensis* (Cyprinidae)	Morocco	OR773478	Rahmouni et al. (2023)
*Gyrodactylus nemachili*	*Oreoleuciscus potanini* (Leuciscidae)	Mongolia	OQ641771	Lebedeva et al. (2023)
*Gyrodactylus nordmanni*	*Oreoleuciscus humilis* (Leuciscidae)	Mongolia	OQ641779	Lebedeva et al. (2023)
*Gyrodactylus papernai*	*Salmo salar* (Salmonidae)	Russia	EF446729	Ziętara et al. (2008)
*Gyrodactylus pseodomoroccensis*	*Luciobarbus ksibi* (Cyprinidae)	Morocco	OR773479	Rahmouni et al. (2023)
*Gyrodactylus pseudonemacheili*	*Thymallus brevirostris* (Salmonidae)	Mongolia	OQ641756	Lebedeva et al. (2023)
*Gyrodactylus rysavyi*	*Clarias gariepinus* (Clariidae)	Senegal	FR850679	Přikrylová et al. (2012b)
*Gyrodactylus salaris*	*Thymallus thymallus* (Salmonidae)	Finland	AF484544	Ziętara and Lumme (2002)
*Gyrodactylus sandai*	*Cyprinoion macrostomum* (Cyprinidae)	Iraq	OR773089	Benovics et al. (2024)
***Gyrodactylus serrai* n. sp.**	***Cheilobarbus serra* (Cyprinidae)**	**South Africa**	**PP800215**	Present study
*Gyrodactylus tayshirensis*	*Barbatula conilobus* (Nemacheilidae)	Mongolia	OQ641774	Lebedeva et al. (2023)
*Gyrodactylus vukicae*	*Garra rufa* (Cyprinidae)	Iraq	OR773090	Benovics et al. (2024)
*Gyrodactylus zavkhanensis*	*Thymallus brevirostris* (Salmonidae)	Mongolia	OQ641773	Lebedeva et al. (2023)
*Gyrodactylus* sp. 1	*Aulopyge huegelii* (Cyprinidae)	Bosnia and Herzegovina	KY848504	Unpublished
*Gyrodactylus* sp. 2	*Carassius auratus* (Cyprinidae)	China	ON117567	Unpublished
*Gyrodactylus* sp. 3	*Carassius auratus* (Cyprinidae)	China	ON117568	Unpublished
*Gyrodactylus* sp. 4	*Luciobarbus bocagei* (Cyprinidae)	Portugal	OR807835	Rahmouni et al. (2023)
*Gyrodactylus* sp. 5	*Luciobarbus comizo* (Cyprinidae)	Spain	OR807837	Rahmouni et al. (2023)
*Gyrodactylus* sp. 6	*Luciobarbus bocagei* (Cyprinidae)	Spain	OR807838	Rahmouni et al. (2023)
*Gyrodactylus* sp. 7	*Labeobarbus maroccanus* (Cyprinidae)	Morocco	OR807839	Rahmouni et al. (2023)
*Gyrodactylus* sp. 8	*Luciobarbus zayanensis* (Cyprinidae)	Morocco	OR807840	Rahmouni et al. (2023)
*Gyrodactylus* sp. 9	*Luciobarbus rabatensis* (Cyprinidae)	Morocco	OR807841	Rahmouni et al. (2023)
*Gyrodactylus* sp. 10	*Luciobarbus massaensis* (Cyprinidae)	Morocco	OR807842	Rahmouni et al. (2023)
*Gyrodactylus* sp. 11	*Luciobarbus rifensis* (Cyprinidae)	Morocco	OR807843	Rahmouni et al. (2023)
*Gyrodactylus* sp. 12	*Luciobarbus yahyauii* (Cyprinidae)	Morocco	OR807844	Rahmouni et al. (2023)
*Gyrodactylus* sp. 13	*Luciobarbus massaensis* (Cyprinidae)	Morocco	OR807845	Rahmouni et al. (2023)
*Gyrodactylus* sp. 14	*Alburnus sellal* (Leuciscidae)	Iraq	OR773084	Benovics et al. (2024)
*Gyrodactylus* sp. 15	*Barbus lacerta* (Cyprinidae)	Iraq	OR773086	Benovics et al. (2024)

The newly generated sequence is presented in bold

Based on the corrected AIC, the generalised time-reversible model, GTR+F+I+G4 (G = 0.26) (Tavaré, 1986; Rodriguez et al., 1990), was selected. The phylogenetic analysis applying maximum likelihood (ML) was computed in IQ-TREE v.2.2. (Nguyen et al., 2015) with bootstrap support values (1000 replicates). A Bayesian Inference (BI) tree was generated in MrBayes v.3.2 (Huelsenbeck and Ronquist, 2001; Ronquist and Huelsenbeck, 2003). Posterior probabilities were calculated using the Metropolis-coupled Markov chain Monte Carlo algorithm (MCMC) for 3× 10^6^ generations, sampling trees every 10^2^ generations. The first 30% of trees were discarded as a relative burn-in period after verifying that the standard deviation split frequency fell below 0.01. The uncorrected *p*-distances and numbers of base pair differences in the alignment were generated using MEGA X (Kumara et al., 2018). Conversion of alignment files was carried out using ALTER v.1.2 (Glez-Peña et al., 2010), and resulting trees were visualised in FigTree v.1.3 (http://tree.bio.ed.ac.uk/software/figtree) and edited using Adobe Illustrator software.

## Results

**Fig. 1:**
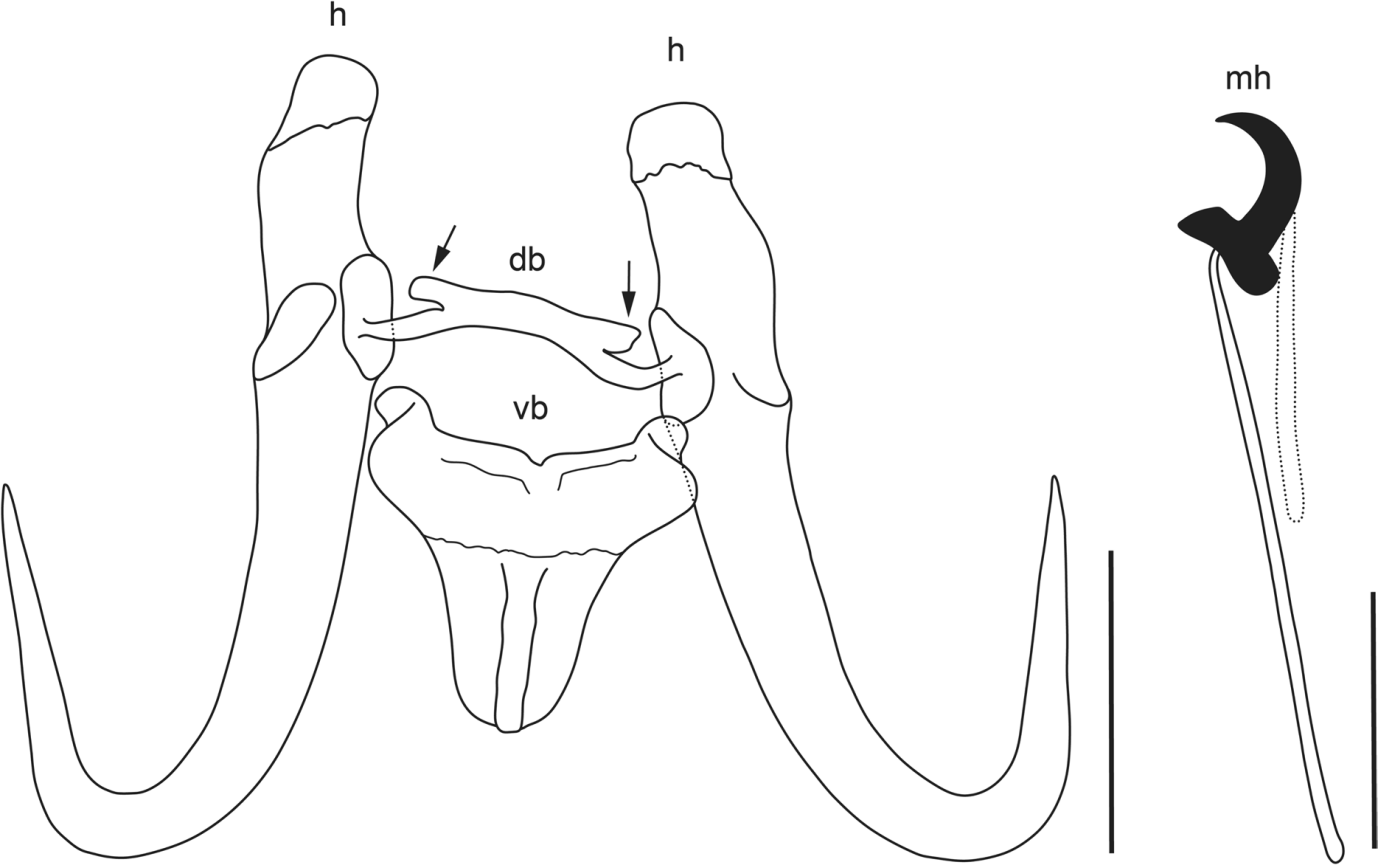
Line drawings of the complex of sclerotised haptoral structures of *Gyrodactylus serrai* **n. sp.** from *Cheilobarbus serra*; h—hamuli, db—dorsal bar, vb—ventral bar, mh—marginal hook. Scale bars = 10 μm.

**Fig. 2:**
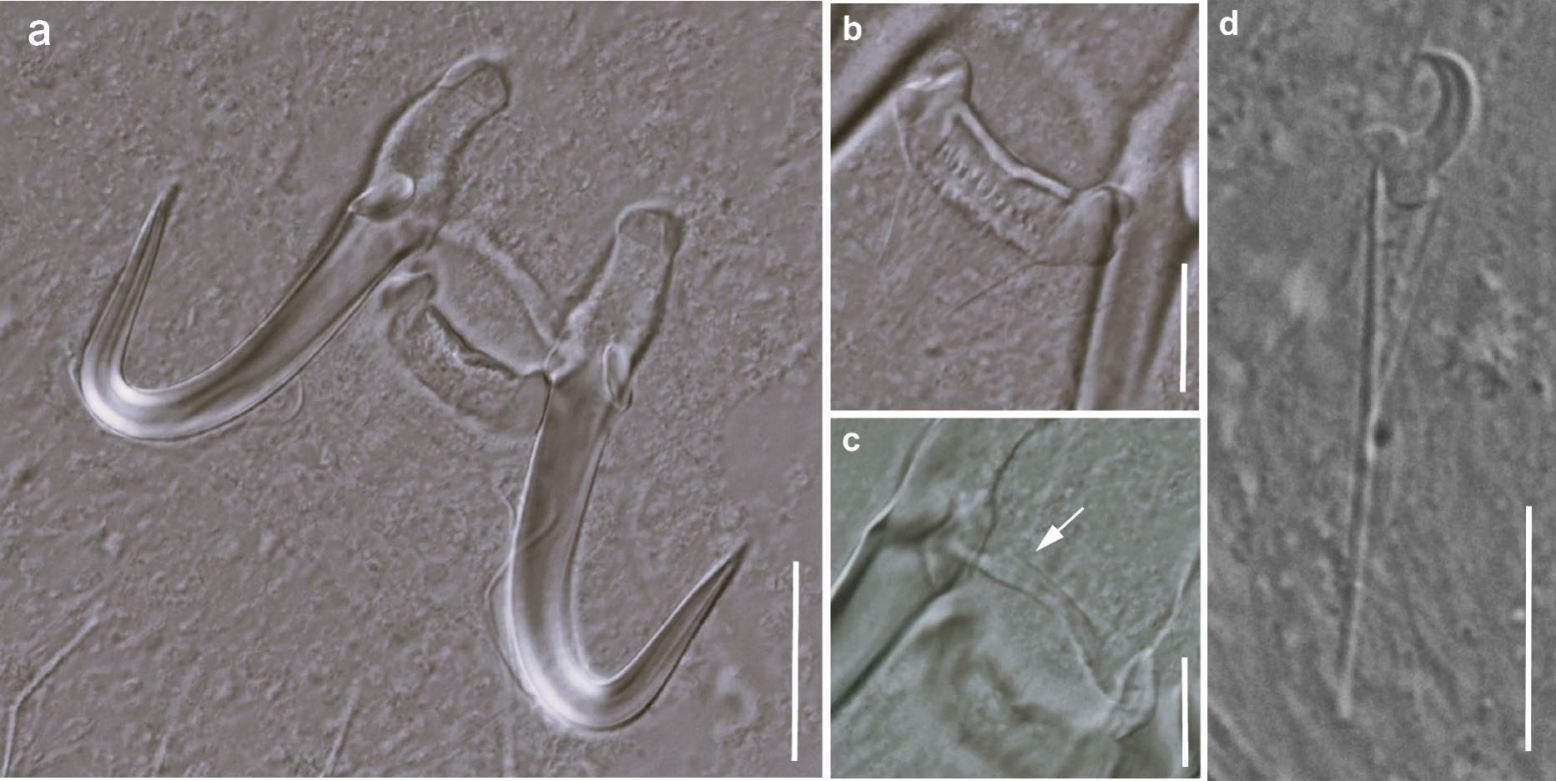
Photomicrographs of *Gyrodactylus serrai* **n. sp.** ex *Cheilobarbus serra*. **A** Complex of sclerotised haptoral structures; **B** Ventral bar; **C** Dorsal bar; **D** Marginal hook. Scale bars: A = 20 μm, B, C, D = 10 μm.

**Fig. 3:**
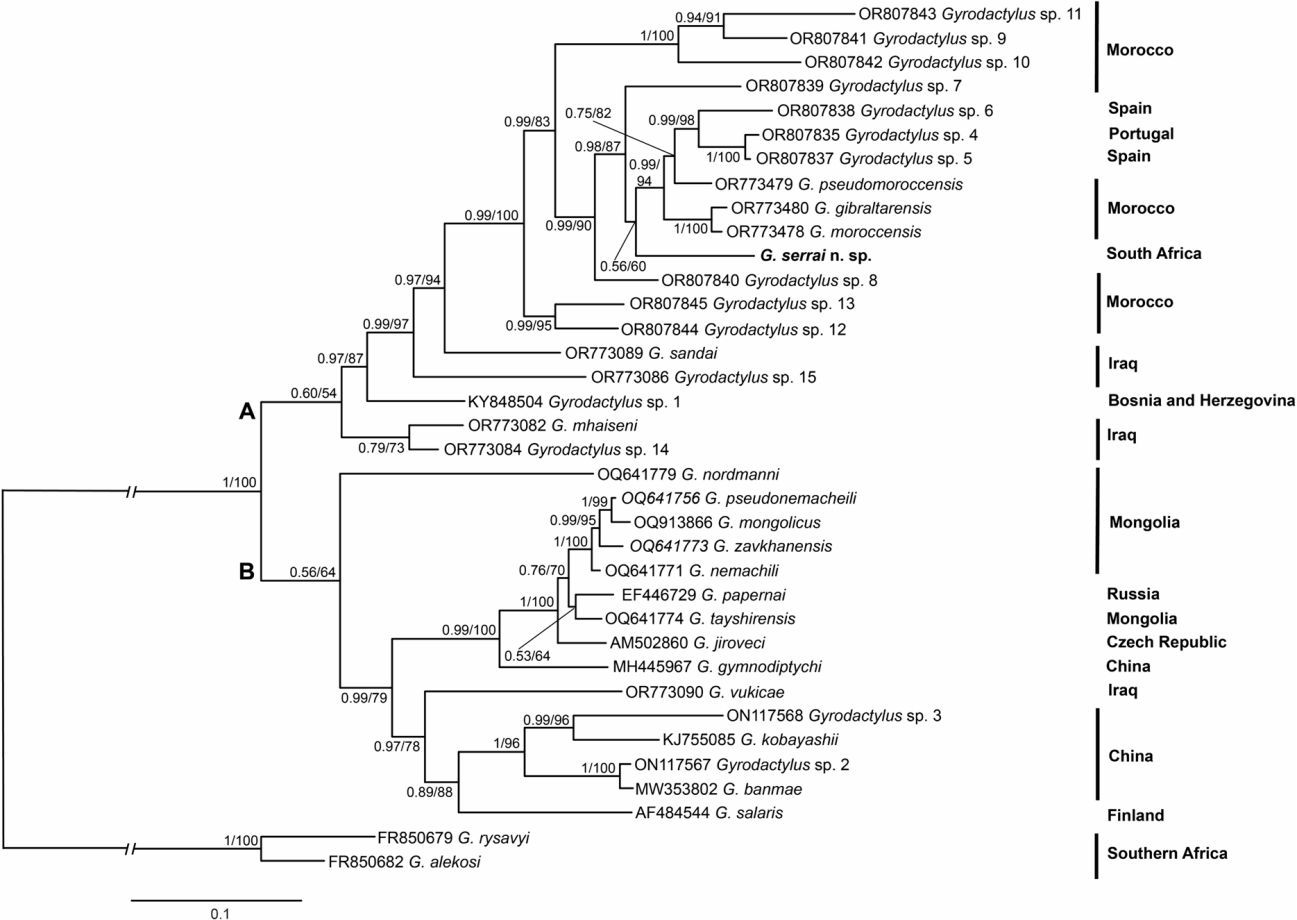
Phylogenetic tree based on the 889 bp alignment of the ITS rDNA sequences of 36 *Gyrodactylus* spp. Values at the nodes indicate posterior probabilities from BI and bootstrap values from ML analysis analyses. Dashes indicate values below 50. Letters (**A**, **B**) represent two well-supported clades. The newly identified species from the present study is in bold.

The gills of five *C. serra* (*P* = 33.3 %) were parasitised with one to two specimens of *Gyrodactylus*. Morphometric and molecular examination of the individuals indicated that these newly collected specimens were morphologically and genetically different from any currently known species of *Gyrodactylus* (Figs. 1, 2, 3; Table 2). The description of the new species is presented below. The present results form part of a larger study and the records of the other parasitic taxa isolated from *C. serra* are presented in Truter et al. (2023).

**Table 2:** Uncorrected p-distance (lower diagonal) and no. of differences (upper diagonal) based on 889 bp ITS rDNA sequences alignment of the species included in the phylogenetic analysis

	Species	1	2	3	4	5	6	7	8	9	10	11	12	13	14	15	16	17	18
1	*G. serrai* n. sp.		264	163	81	159	167	161	135	161	77	160	167	164	78	162	267	172	121
2	*G. alekosi*	33.5		260	262	254	265	265	243	264	263	264	245	268	265	263	60	263	252
3	*G. banmae*	18.4	33.0		168	139	143	92	146	145	169	147	161	147	163	150	267	116	151
4	*G. gibraltarensis*	9.2	33.3	18.9		157	162	160	124	164	11	161	159	161	43	166	262	165	113
5	*G. gymnodiptychi*	17.9	32.3	15.7	17.7		84	141	143	84	154	85	134	86	150	88	265	134	149
6	*G. jiroveci*	18.9	33.7	16.1	18.3	9.5		141	148	49	158	36	147	43	157	43	263	126	169
7	*G. kobayashii*	18.2	33.6	10.3	18.0	15.9	15.9		142	136	157	137	143	133	161	139	267	121	159
8	*G. mhaiseni*	15.2	30.8	16.4	14.0	16.1	16.7	16.0		142	124	142	140	146	125	146	251	147	109
9	*G. mongolicus*	18.2	33.5	16.3	18.5	9.5	5.5	15.3	16.0		162	19	138	43	159	10	265	121	158
10	*G. moroccensis*	8.7	33.5	19.0	1.2	17.4	17.8	17.7	14.0	18.3		157	155	158	43	162	263	166	107
11	*G. nemachili*	18.1	33.5	16.5	18.1	9.6	4.1	15.4	16.0	2.1	17.7		138	31	155	13	263	122	155
12	*G. nordmanni*	19.0	31.5	18.3	18.1	15.2	16.8	12.3	15.9	15.7	17.7	15.7		147	153	142	252	156	150
13	*G. papernai*	18.5	34.0	16.5	18.1	9.7	4.8	15.0	16.4	4.8	17.8	3.5	16.7		157	39	263	124	160
14	*G. pseudomoroccensis*	8.8	33.6	18.3	4.8	16.9	17.7	18.1	14.1	17.9	4.8	17.5	17.4	17.7		159	267	172	110
15	*G. pseudonemacheili*	18.3	33.4	16.9	18.7	9.9	4.8	15.6	16.5	1.1	18.3	1.4	16.2	4.4	17.9		265	124	158
16	*G. rysavyi*	33.6	7.6	33.6	33.0	33.4	33.2	33.6	31.6	33.3	33.2	33.1	31.1	33.1	33.6	33.4		268	260
17	*G. salaris*	19.4	33.4	13.1	18.6	15.1	14.2	13.6	16.6	13.6	18.7	13.7	17.8	13.9	19.4	14.0	33.7		168
18	*G. sandai*	13.7	32.0	17.0	12.8	16.8	19.1	17.9	12.3	17.8	12.1	17.5	17.1	18.0	12.4	17.8	32.8	19.0	
19	*G. tayshirensis*	17.8	34.3	15.4	18.1	8.8	4.3	14.8	16.2	3.9	17.6	3.1	15.6	3.4	17.3	3.9	33.3	13.2	17.6
20	*G. vukicae*	18.8	32.7	14.7	19.7	16.6	16.5	14.6	15.7	16.4	19.6	16.8	18.0	17.0	18.8	16.6	33.4	14.7	18.1
21	*G. zavkhanensis*	18.9	33.2	16.8	18.8	9.7	4.5	15.6	16.7	2.6	18.4	2.0	15.9	4.3	18.0	1.8	32.9	13.5	17.6
22	*Gyrodactylus* sp. 1	15.1	31.7	15.9	15.0	15.7	17.4	15.0	9.9	15.9	14.4	16.7	16.6	16.3	14.8	16.4	32.3	16.0	12.1
23	*Gyrodactylus* sp. 2	18.3	33.2	1.2	19.2	15.2	15.8	10.2	16.3	16.0	19.2	16.2	17.6	16.3	18.6	16.5	33.8	12.7	16.9
24	*Gyrodactylus* sp. 3	18.4	33.9	12.5	19.6	17.0	18.0	10.7	16.8	17.8	19.4	17.8	18.0	18.2	19.3	18.2	34.6	15.7	18.7
25	*Gyrodactylus* sp. 4	9.8	33.7	17.7	7.1	17.7	18.0	17.3	14.6	18.0	6.7	17.9	18.9	18.2	5.6	17.8	33.8	17.9	13.0
26	*Gyrodactylus* sp. 5	9.1	33.4	17.4	6.5	17.5	17.8	17.4	14.1	17.9	6.2	17.7	19.0	18.2	5.2	17.7	33.6	18.0	12.5
27	*Gyrodactylus* sp. 6	9.9	33.0	18.7	7.3	18.4	19.8	18.6	15.5	19.4	7.1	19.2	18.8	19.2	6.1	19.4	33.8	19.6	13.4
28	*Gyrodactylus* sp. 7	11.2	33.5	19.5	8.9	19.3	20.7	18.2	15.9	20.2	8.4	20.0	18.9	19.9	8.8	20.1	33.6	20.0	13.9
29	*Gyrodactylus* sp. 8	9.4	33.2	18.3	7.9	16.9	18.5	16.7	14.1	18.1	7.2	17.5	17.5	17.0	7.5	17.9	33.3	17.9	11.7
30	*Gyrodactylus* sp. 9	14.7	32.5	19.6	13.7	18.6	19.0	18.0	16.5	18.4	13.3	17.8	18.8	18.4	12.8	18.1	33.4	19.5	14.4
31	*Gyrodactylus* sp. 10	14.4	32.9	19.5	13.3	17.7	18.2	18.4	16.4	18.1	12.5	17.8	18.6	18.0	13.0	18.2	33.6	19.6	14.0
32	*Gyrodactylus* sp. 11	16.2	32.2	20.3	13.9	18.6	18.2	18.5	16.2	18.3	13.6	17.6	19.3	18.1	13.3	18.0	33.0	19.2	14.4
33	*Gyrodactylus* sp. 12	12.7	32.2	18.5	10.1	17.1	18.7	16.8	13.3	17.6	10.0	17.9	18.2	17.9	10.1	18.2	32.5	18.8	11.4
34	*Gyrodactylus* sp. 13	12.3	33.5	17.5	10.4	17.5	18.6	16.0	13.9	18.0	10.2	17.6	18.0	16.8	9.8	18.3	33.5	18.2	11.7
35	*Gyrodactylus* sp. 14	14.9	30.4	16.2	14.6	15.2	16.7	15.0	4.3	15.3	14.2	15.5	15.5	16.3	13.8	15.7	31.6	15.9	12.2
36	*Gyrodactylus* sp. 15	16.5	33.4	19.2	15.7	18.4	19.2	18.5	13.0	18.3	15.2	18.2	19.5	18.5	15.8	18.5	33.5	19.4	11.8

	Species	19	20	21	22	23	24	25	26	27	28	29	30	31	32	33	34	35	36
1	*G. serrai* n. sp.	158	166	168	134	162	163	87	81	87	99	83	130	126	142	112	109	132	145
2	*G. alekosi*	270	267	262	250	262	267	266	263	258	264	260	255	255	250	252	264	239	261
3	*G. banmae*	137	130	149	141	11	111	157	155	165	173	162	173	170	178	163	155	144	170
4	*G. gibraltarensis*	161	174	167	133	170	173	63	58	64	79	70	121	116	122	89	92	129	138
5	*G. gymnodiptychi*	78	147	86	139	135	150	157	155	162	171	149	164	154	163	151	155	135	162
6	*G. jiroveci*	38	146	40	154	140	159	160	158	174	184	163	168	159	159	165	164	148	169
7	*G. kobayashii*	132	129	139	133	91	95	154	155	164	162	148	159	161	162	148	142	133	163
8	*G. mhaiseni*	144	139	148	88	145	149	130	125	137	141	125	146	143	142	117	123	38	115
9	*G. mongolicus*	35	145	23	141	142	158	160	159	171	180	160	163	158	161	155	159	136	162
10	*G. moroccensis*	156	173	163	128	170	172	60	55	63	75	64	117	109	119	88	90	126	134
11	*G. nemachili*	28	149	18	148	144	158	158	157	169	178	155	157	155	154	158	156	137	161
12	*G. nordmanni*	137	158	140	146	155	158	166	167	164	166	153	165	161	167	159	158	136	170
13	*G. papernai*	30	151	38	145	145	161	162	162	169	177	150	163	157	159	158	149	145	163
14	*G. pseudomoroccensis*	154	167	160	132	165	171	50	46	54	78	66	113	114	117	89	87	123	140
15	*G. pseudonemacheili*	35	147	16	146	147	161	158	157	171	179	158	160	159	158	160	162	139	163
16	*G. rysavyi*	265	265	262	257	269	275	269	267	267	267	263	264	263	259	256	266	251	264
17	*G. salaris*	117	130	120	142	113	139	159	160	173	178	158	172	171	168	166	161	141	171
18	*G. sandai*	156	160	156	107	150	165	115	111	118	123	103	127	122	126	100	103	108	104
19	*G. tayshirensis*		144	35	146	134	155	157	156	168	180	152	158	151	155	162	163	142	162
20	*G. vukicae*	16.3		146	139	135	129	164	162	176	177	156	169	167	179	166	165	131	153
21	*G. zavkhanensis*	3.9	16.5		147	146	160	160	159	172	182	158	162	156	157	159	156	142	164
22	*Gyrodactylus* sp. 1	16.4	15.7	16.5		141	145	137	131	142	140	120	134	127	140	108	119	84	109
23	*Gyrodactylus* sp. 2	15.1	15.2	16.4	15.8		113	160	158	167	173	162	175	169	178	164	154	146	170
24	*Gyrodactylus* sp. 3	17.5	14.6	18.1	16.4	12.7		172	173	184	171	160	164	171	173	157	148	142	174
25	*Gyrodactylus* sp. 4	17.7	18.5	18.0	15.4	18.0	19.4		8	56	90	76	114	117	124	98	96	129	140
26	*Gyrodactylus* sp. 5	17.5	18.3	17.9	14.7	17.8	19.5	0.9		54	86	77	114	115	126	98	95	123	134
27	*Gyrodactylus* sp. 6	19.0	20.0	19.5	16.1	18.9	20.9	6.3	6.1		90	76	118	121	128	93	95	136	147
28	*Gyrodactylus* sp. 7	20.2	20.0	20.5	15.7	19.5	19.3	10.1	9.7	10.2		75	129	130	136	101	96	137	153
29	*Gyrodactylus* sp. 8	17.2	17.7	17.9	13.6	18.3	18.2	8.6	8.7	8.7	8.4		106	101	112	77	78	119	132
30	*Gyrodactylus* sp. 9	17.9	19.2	18.3	15.1	19.8	18.6	12.9	12.9	13.4	14.4	12.0		78	74	108	115	146	146
31	*Gyrodactylus* sp. 10	17.3	19.2	17.9	14.5	19.3	19.6	13.0	13.2	14.0	14.9	11.6	8.9		100	110	110	144	139
32	*Gyrodactylus* sp. 11	17.7	20.5	17.9	15.9	20.3	19.8	14.1	14.4	14.7	15.5	12.8	8.4	11.5		115	122	150	149
33	*Gyrodactylus* sp. 12	18.4	18.9	18.0	12.2	18.6	17.8	11.1	11.1	10.6	11.5	8.8	12.3	12.7	13.2		54	112	125
34	*Gyrodactylus* sp. 13	18.4	18.7	17.6	13.4	17.4	16.8	10.8	10.7	10.8	10.8	8.8	13.1	12.6	14.0	6.1		117	124
35	*Gyrodactylus* sp. 14	16.0	14.8	16.0	9.5	16.5	16.1	14.5	13.9	15.4	15.4	13.5	16.5	16.5	17.0	12.7	13.2		110
36	*Gyrodactylus* sp. 15	18.3	17.4	18.6	12.3	19.2	19.9	15.8	15.2	16.8	17.3	15.0	16.6	16.0	17.2	14.3	14.1	12.5	

The newly generated sequence is presented in bold

**Gyrodactylidae van Beneden & Hesse, 1832**

***Gyrodactylus*** **von Nordmann, 1832**

***Gyrodactylus serrai*** **n. sp.**

*Type-host*: *Cheilobarbus serra* (Peters) (Cyprinidae, Smiliogastrinae)

*Type locality:* Matjies River (32º30’00” S, 19º19’57” E), Cederberg Wilderness Area, Western Cape Province, South Africa

*Type-material*: Holotype (NMB P 1036), two paratypes (NMB P 1037 and 1038) and one hologenophore (NMB P 1039), two paratypes IPBCAS (M – 797).

*Site of infections*: Gill filaments.

*ZooBank registration*: urn:lsid:zoobank.org:act:247315F9-F25A-4432-9AC6-59ABD80BAF08.

*Synonym*: *Gyrodactylus* sp. *sensu* Truter et al. (2023)

*DNA sequence*: A nucleotide sequence of ITS1-5.8S-ITS2 rDNA region (1012 bp; access. No PP800215).

*Etymology*: The specific name is derived from the species name of the type-host, *Cheilobarbus serra.*

Description (Figs. 1, 2)

[based on 8 specimens in AGP and 1 hologenophore]. Overall body appearance as defined for the genus as determined in Gussev (1985) and Řehulková et al. (2018). Total body length of coverslip flattened specimens 575–824 (698.5;* n* = 4); width 106–201 (159.5, *n* = 4) at level of uterus. Pharyngeal bulb width 30.4–35 (32.8, *n* = 3) and length 15–23.8 (19.8, *n* = 3) across anterior bulb; width 35.7–45.6 (42, *n* = 4) and length 21.2–35 (27.9, *n* = 4) across posterior bulb. Male copulatory organ (MCO) 14.4–20.2 (17, *n* = 3) in diameter, composition of spines not observable. Up to two developing embryos observed in four specimens.

Hamuli with well-developed inner roots; roots facing upwards and turn in their mid-length slightly internally, narrow towards the end of roots (Fig. 2A). Hamuli shaft turns into point with tip projecting nearly straight upwards and reaches near to 2/3 of hamuli shaft length. Hamuli total length 43.2–50.3 (47.2, *n* = 9), inner root length 11.7–16.7 (14, *n* = 9), hamuli shaft length 31.9–37.5 (34.6, *n* = 9) and hamuli point length 20.7–23.9 (22.4, *n* = 9). Hamuli connected with dorsal bar, branch-like projections on upper part of bar [Figs 1 (db), 2C)] 17.8–21 (19, *n* = 9) wide, 1.4–2.4 (2, *n* = 9) long. Ventral bar bears tongue-like shaped membrane with well-developed terminal ridge and having short lateral processes, body of ventral bar with depression in middle of upper line [Figs. 1 (vb), 2B]. Ventral bar median length 5–6.4 (5.6, *n* = 9), membrane length 10.2–13.2 (12.2, *n* = 9), ventral bar width 19.1–22.7 (20.6, *n* = 9), lateral processes 1.2–2.2 (1.7, *n* = 9) long. Marginal hook total length 25.1–27.8 (26.5, *n* = 8), marginal hook shaft length 20.1–22.2 (21.2, *n* = 11), marginal hook proper 4.9–5.3 (5.1, *n* = 12) long, marginal hook proper proximal width 3.5–4.1 (3.8, *n* = 10), marginal hook distal width 3.3–3.6 (3.5, *n* = 9). Sickle proper well separated, rises slightly forward from middle part of base, turns gradually into point. Point projecting slightly downwards and ends beyond tip of toe of sickle proper. Pronounced sickle heel, slightly rounded. Upper line of toe flat, in half of its length slopes into blunt tip that heads moderately downwards [Figs. 1 (mh), 2D].

### Remarks

Of the six *Gyrodactylus* spp. known from the African continent from cyprinid hosts, *G. serrai* **n. sp.** is similar in the overall shape of hamuli and ventral bar to *G. ivindoensis* ex *Enteromius holotaenia* (Boulenger) from Gabon and *G. nyingiae* ex *Luciobarbus pallaryi* (Pellegrin) from Morocco. *G. serrai* **n. sp.** can be differentiated from both species: (1) by having smaller hamuli, total hamuli length of *G. serrai* **n. sp.** 43.2–50.3 vs *G. ivindoensis* 52–58 and *G. nyingiae* 65.9–88.8; hamulus point length *G. serrai* **n. sp.** 20.7–23.9 vs *G. nyingiae* 31.7–42.3; (2) by having less pronounced lateral processes of the ventral bar and a smaller width of the ventral bar, *G. serrai* **n. sp.** ventral bar width 19.1–22.7 vs *G. ivindoensis* 27.1 and *G. nyingiae* 24.8–25.4; (3) size and shape of the marginal hook, the total length of marginal hook *G. serrai* **n. sp.** 25.1–27.8 vs *G. ivindoensis* 21–24 and *G. nyingiae* 31.7–42.1. Additionally, the marginal hook sickle proper of *G. ivindoensis* is more subtle, sickle widely opened with point heading upwards and not reaching beyond the tip of the toe, while the marginal hook sickle of *G. serrai* **n. sp.** is more sturdy, curving gradually into a point which ends beyond the tip of the toe. *Gyrodactylus serrai* **n. sp.** is distinguishable from the four remaining *Gyrodactylus* spp. known on cyprinid hosts (*G. kyogae*, *G. moroccensis*, *G. paludinosus*, *G. pseudomoroccensis*) by the overall appearance of the haptoral hard structures.

### Molecular Characterisation and Phylogenetic Reconstruction

The ITS1-5.8S-ITS2 rDNA regions for one out of three isolates were successfully amplified and sequenced. The haptor of the sequenced specimens is deposited as hologenophore (for accession number see above). The sequence of 1012 bp consists of partial ITS1 (469 bp), 5.8S (157 bp), and partial ITS2 (385 bp) rDNA and the final alignment consisted of 36 *Gyrodactylus* spp., including *G. alekosi* and *G. rysavyi* as the outgroup, spanned 1320 bp before trimming, and the final trimmed alignment of unambiguously aligned sequences used in the analysis was 889 bp.

The ML and BI analyses generated trees with identical topologies. The phylogenetic analysis (Fig. 3) divided species into two well-supported lineages. *Gyrodactylus serrai* **n. sp.** clustered within lineage A that includes species of *Gyrodactylus* from peri-Mediterranean countries (Bosnia and Herzegovina, Morocco, Portugal, and Spain) and Iraq. Within lineage A, *G. serrai* **n. sp.** forms part of a well-supported cluster, that has an early divergent sub-lineage formed by *Gyrodactylus* sp. 12 and *Gyrodactylus* sp. 13, both from Moroccan *Luciobarbus* spp., the other three species of the same origin and host genus (*Gyrodactylus* spp. 9-11) clustered in a separate well-supported sub-lineage. *Gyrodactylus serrai* **n. sp.** resulted as a sister taxon to the species in a highly supported cluster formed by four species known from *Luciobarbus* spp. from the Iberian Peninsula (*G. gibraltarensis* and *Gyrodactylus* spp. 4-6) and two species, *G. moroccensis* and *G. pseudomoroccensis* from Moroccan hosts of *Luciobarbus.* Within lineage B, the position of *Gyrodactylus nordmanni* Ergens & Dulmaa, 1970 remains unresolved, while other species are split into two sub-lineages. One lineage is formed by seven congeners of various origins (China, Czech Republic, Mongolia, and Russia) and hosts of Leuciscidae, Nemacheilidae and Salmonidae. The second sub-lineage, not fully resolved, is formed by five *Gyrodactylus* spp. from host species of Cyprinidae from China and Iraq, and *Gyrodactylus salaris* Malmberg, 1957.

Based on the uncorrected *p*-distances, the most closely related species to *G. serrai* **n. sp.** appeared to be *G. moroccensis* and *G. pseudomoroccensis*, 8.7 and 8.8%, respectively (Table 2). Other species with a *p*-distances below 10% between them and the newly described species are *G. gibraltarensis* (9.2%), *Gyrodactylus* spp. 4-5 (9.1–9.9%) and *Gyrodactylus* sp. 8 (9.4%). The lowest *p*-distance was observed between *Gyrodactylus pseudonemacheili* Ergens & Bychowsky, 1967 and *Gyrodactylus zavkhanensis* Lebedeva et al., 2023 (1.1%). The group of species, *Gyrodactylus jiroveci* Ergens & Bychowsky, 1967, *Gyrodactylus monoglicus* Lebedeva et al. 2023, *Gyrodactylus nemachili* Bychowsky, 1936*, Gyrodactylus papernai* Ergens & Bychowsky, 1967, *G. pseudonemacheili*, *Gyrodactylus tayshirensis* Lebedeva et al. 2023 and *G. zavkhanensis*) represents closely related species (1.1–4.8%).

## Discussion

The description of *G. serrai* **n. sp.** from an endemic cyprinid host, *C. serra*, makes this the fourth species of this genus formally described from a freshwater host in South Africa. This is also the first parasite to be described from a species in the genus *Cheilobarbus* (Řehulková et al., 2018; Truter et al., 2022). *Gyrodactylus serrai* **n. sp.** is easily separated from congeners (*G. kyogae*, *G. moroccensis*, *G. paludinosus*, and *G. pseudomoroccensis*) by differences in the morphology of the haptoral sclerites (Figs. 1 and 2) as well as differences in host preference. The ventral bar of the newly identified species *G. serrai* **n. sp.** has a well-developed ridge through most of the length of its membrane. This feature is seen in Eurasian and Mediterranean morphotypes and provide support to the Asian origin of hosts in the Smiliogastrinae and its associated parasites, which most likely dispersed into Africa through a single event and subsequent diversification and dispersal. This allowed for parasitic species to develop differentiating features, while retaining morphological similarities with common ancestors (Gussev, 1985; Yang et al., 2015; Ren and Mayden, 2016; Lavoué, 2020; Rahmouni et al., 2023).

Molecular inference with congeners provided insights into the position of *G. serrai* **n. sp.** by placing it in a well-supported lineage consisting of gyrodactylids from various cyprinoid species from Iraq and peri-Mediterranean countries (Fig. 3, lineage A). In Africa, there are most probably still many taxa to be discovered and as a consequence the phylogenies are partial, and the currently available data can’t support a strong conclusive interpretation. Our phylogenetic analyses confirm this in clusters within lineage A which are well-supported, however, high values of uncorrected *p*-distances indicate that closely related species data is not available for South African species, as those could be species with *p*-distances between 2 and 5%. In our analysis, two species within lineage B, *G. pseudonemacheili* and *G. zavkhanensis* are the most closely related, with the lowest observed *p*-distance of 1.1%, while the closest species to *G. serrai* **n. sp.** were the Moroccan species, *G. moroccensis* and *G. pseudomoroccensis*, 8.7 and 8.8%, respectively. Compared to other studies that focus on African *Gyrodactylus* species, genetic data availability provides an understanding of the evolutionary relationships between species from hosts within the same families or speciation events which is currently not possible for gyrodactylids of African cyprinoids (see Přikrylová et al. 2012a, 2012b; Rahmouni et al. 2023). We encourage continued efforts to obtain and supplement species descriptions with molecular data, as there is only comparative data available for 16 African gyrodactylids, all primarily known from cichlids and catfishes (Přikrylová et al., 2013; Christison et al., 2021; Rahmouni et al., 2023).

Although the application of molecular techniques in species descriptions is not always successful or possible to complement morphological descriptions (see Přikrylová et al., 2017; Raphahlelo et al., 2020; Truter et al., 2022), careful examination and visual documentation in the form of photomicrographs and detailed drawings should be provided to eliminate taxonomic discrepancies. This is especially important where, what is most probably two separate species, is presented as a single species, as can be seen in Shigoley et al. (2023). Similarly, clarity and resolution of species relationships will be promoted with better availability of genetic data.

Despite the vast diversity of cyprinid hosts across the African continent, only seven *Gyrodactylus* species parasitising cyprinid hosts from four African countries are known (Řehulková et al., 2018; Truter et al., 2022; Rahmouni et al., 2023; Shigoley et al., 2023; present study). Until recently, *Gyrodactylus* from cyprinid hosts in Africa were represented by only *G. kyogae* and *G. ivindoensis* from Uganda and Gabon in central Africa, respectively. Nearly 50 years after the description of the aforementioned species, *G. paludinosus* was discovered from a small cyprinid in South Africa and another three from large cyprinids in Morocco (Truter et al., 2022; Rahmouni et al., 2023; Shigoley et al., 2023). Considering known fish diversity and discovery over time, the estimate of gyrodactylid diversity is between 10,000 and 20,000 species in diverse ecosystems worldwide (Bakke et al., 2002).

The description of *G. serrai* **n. sp.** is a result of the investigation of the indigenous cyprinid hosts in South Africa’s Cape Fold Ecoregion (Truter et al., 2023), a unique area that is characterised by high endemism (Chakona et al., 2022). Although South Africa does not house an immense fish diversity as found in tropical countries, the present endemic hosts in its freshwater systems are important objects to study to illustrate and document its unique fish parasites. These parasites that have over decades of interest in the hosts’ taxonomy, biology and conservation never been considered as a contributing component to endemic biodiversity, and we therefore advocate here for increased attention and study of these fish species that can aid in improved efforts in conserving highly endemic hosts and their idiosyncratic parasites.

## Data Availability

No datasets were generated or analysed during the current study.

## References

[CR2] Acosta, A. A., Truter, M., Malherbe, W, & Smit, N. J. (2022). Morphological description and molecular characterisation of *Dactylogyrus matlopong* sp. n. (Monogenea: Dactylogyridae) from the South African endemic *Labeobarbus aeneus* (Cyprinidae: Torinae). *Folia Parasitologica, 69*, 021. 10.14411/fp.2022.021 10.14411/fp.2022.021 36227137

[CR3] Bakke, T. A., Harris, P. D., & Cable, J. (2002). Host specificity dynamics: Observations on gyrodactylid monogeneans. *International Journal for Parasitology, 32*, 281–308. 10.1016/s0020-7519(01)00331-9 11835970 10.1016/s0020-7519(01)00331-9

[CR4] Benovics, M., Rahmouni, C., Řehulková, E., Nejat, F., & Šimková, A. (2024). Uncovering the monogenean species diversity of cyprinoid fish in Iraq using an integrative approach. *Parasitology 151,* 220–246. 10.1017/S0031182023001348 38116665 10.1017/S0031182023001348 PMC10941050

[CR5] Capella-Gutiérrez, S., Silla-Martínez, J. M., & Gabaldón, T. (2009). TrimAl: a tool for automated alignment trimming in large-scale phylogenetic analyses. *Bioinformatics, 25*, 1972–1973. 10.1093/bioinformatics/btp348 19505945 10.1093/bioinformatics/btp348 PMC2712344

[CR6] Chakona, A., Swartz, E. R., & Gouws, G. (2013). Evolutionary drivers of diversification and distribution of a southern temperate stream fish assemblage: Testing the role of historical isolation and spatial range expansion. *PLoS ONE, 8*(8), e70953. 10.1371/journal.pone.0070953 23951050 10.1371/journal.pone.0070953 PMC3739774

[CR7] Chakona, A., Swartz, E. R., & Skelton, P. (2014). A new species of redfin (Teleostei, Cyprinidae, *Pseudobarbus*) from the Verlorenvlei River system, South Africa. *Zookeys, 453,* 121–137. 10.3897/zookeys.453.8072 10.3897/zookeys.453.8072 PMC4258629 25493062

[CR8] Chakona, A., Jordaan, M. S., Raimondo, D. C., Bills, R. I., Skelton, P.H., & Van der Colff, D. (2022). Diversity, distribution and extinction risk of native freshwater fishes of South Africa. *Journal of Fish Biology, 100*, 1044–1061. 10.1111/jfb.15011 35170047 10.1111/jfb.15011

[CR9] Christison, K. W., Shinn, A. P., & Van As. J. G. (2005). *Gyrodactylus thlapi* n. sp. (Monogenea) from *Pseudocrenilabrus philander philander* (Weber) (Cichlidae) in the Okavango Delta, Botswana. *Systematic Parasitology, 60*, 165–173. 10.1007/s11230-004-6342-x 15864454 10.1007/s11230-004-6342-x

[CR10] Christison, K. W., Vaughan, D. B., Shinn, A. P., & Hansen, H. (2021). *Gyrodactylus molweni* sp. n. (Monogenea: Gyrodactylidae) from *Chelon richardsoni* (Smith, 1846) (Mugilidae) from Table Bay, South Africa. *International Journal for Parasitology: Parasites and Wildlife, 15*, 87–94. 10.1016/j.ijppw.2021.02.11 33996440 10.1016/j.ijppaw.2021.02.011 PMC8102207

[CR11] Ergens, R. (1969). The suitability of ammonium picrate-glycerin preparing slides of lower Monogenoidea. *Folia Parasitologica, 16*, 320.

[CR12] Fricke, R., Eschmeyer, W. N., & van der Laan, R. (Eds.). (2023). Eschmeyer’s Catalog of Fishes: genera, species, references. (http://researcharchive.calacademy.org/research/ichthyology/catalog/fishcatmain.asp). (accessed 15 January 2024).

[CR13] García-Vásquez, A., Hansen, H., Christison, K. W., Bron, J. E. & Shinn, A. P. (2011). Description of three new species of *Gyrodactylus* von Nordmann, 1832 (Monogenea) parasitising *Oreochromis niloticus niloticus* (L.) and *O. mossambicus* (Peters) (Cichlidae). *Acta Parasitologica, 56,* 20–33. 10.2478/s11686-011-0005-2

[CR14] García-Vásquez, A., Piancho-Pinacho, C. D., Guzmán-Valdivieso, I., Salgado-Maldomado, G, & Rubio-Godot, M. (2019). New species of *Gyrodactylus* von Nordmann, 1832 from native fish from Chiapas, Mexico, Studied by morphology and molecular analysis. *Acta Parasitologica, 64*, 551–565. 10.2478/s11686-019000088-z 31165989 10.2478/s11686-019-00088-y

[CR15] Glez-Peña, D., Gómez-Blanco, D., Reboiro-Jato, M., Fdez-Riverola, F., & Posada, D. (2010). ALTER: program-oriented conversion of DNA and protein alignments. *Nucleic Acids Research, 38*, W14–W18. 10.1093/nar/gkq321 20439312 10.1093/nar/gkq321 PMC2896128

[CR16] Gussev, V. A. (1985). Identifier of parasites off Freshwater fishes of USSR Fauna. *II. L. Science:* pp 425.

[CR17] Harris, P. D., Shinn, A. P., Cable, J., & Bakke, T. A. (2004). Nominal species of the genus *Gyrodactylus* von Nordmann 1832 (Monogenea: Gyrodactylidae), with a list of principal host species. *Systematic Parasitology, 59*, 1–27. 10.1023/B:SYPA.0000038447.52015.e4 15318017 10.1023/B:SYPA.0000038447.52015.e4

[CR18] Huelsenbeck, J. P., & Ronquist, F. (2001). MrBayes: Bayesian inference of phylogenetic trees. *Bioinformatics, 17*, 754–755. 10.1093/bioinformatics/17.8.754 11524383 10.1093/bioinformatics/17.8.754

[CR19] Hurvich, C. M., Tsai, C. L. (1989). Regression and time series model selection in small samples. *Biometrika, 76*, 297–307. 10.1093/biomet/76.2.297

[CR20] Impson, D., Van der Walt, R., & Jordaan, M. (2017). *Pseudobarbus serra*. The IUCN Red List of threatened pecies 2017: e.T2569A100148283. 10.2305/IUCN. UK.2017-3.RLTS.T2569A100148283.en. (accessed 15 January 2024)

[CR21] Jin, X., Cheng, H., Li, M., Zou, H., Cai, J., Amoah, K., Li, W., & Wang, G. (2024). Description of three new species of *Gyrodactylus* von Nordmann, 1832 (Monogenea: Gyrodactylidae) on bitterling fishes (Acheilognathinae) from China. *Parasitology International, 101*(5), 102893. 10.1016/j.parint.2024.102893 38588816 10.1016/j.parint.2024.102893

[CR22] Jin, X., Li, W., Cheng, Y., Li, M., Wu, S., Zou, H., & Wang, G. (2022). Description of *Gyrodactylus banmae* n. sp. (Monogenea, Gyrodactylidae) parasitic on zebrafish, *Danio rerio*. *Parasitology International, 87,* 102531. 10.1016/j.parint.2021.102532 10.1016/j.parint.2021.102531 34929406

[CR23] Kalyaanamoorthy, S., Minh, B. Q., Wong, T. K. F., von Haeseler, A., & Jermiin, L. S. (2017). ModelFinder: Fast model selection for accurate phylogenetic estimates. *Nature Methods, 14*, 587–589. 10.1038/nmeth.4285 28481363 10.1038/nmeth.4285 PMC5453245

[CR24] Katoh, K., Rozewicki, J., & Yamada, K. D. (2019). MAFFT online service: Multiple sequence alignment, interactive sequence choice and visualization. *Briefings in Bioinformatics, 20*, 1160–1166. 10.1093/bib/bbx108 28968734 10.1093/bib/bbx108 PMC6781576

[CR25] Kearse, M., Moir, R., Wilson, A., Stones-Havas, S., Cheung, M., Sturrock, S., Buxton, S., Cooper, A., Markowitz, S., Duran, C., Thierer, T., Ashton, B., Meintjes, P., & Drummond, A. (2012). Geneious Basic: an integrated and extendable desktop software platform for the organization and analysis of sequence data. *Bioinformatics, 28,* 1647–1649. 10.1093/bioinformatics/bts199 22543367 10.1093/bioinformatics/bts199 PMC3371832

[CR26] Kumara, S, Stecher, G., Li, M., Knyaz, C., & Tamura, K. (2018). MEGA X: Molecular Evolutionary Genetics Analysis across computing platforms. *Molecular Biology and Evolutions, 35*, 1547–1549. 10.1093/molbev/msy096 10.1093/molbev/msy096 PMC5967553 29722887

[CR27] Lavoué, S. (2020). Origins of Afrotropical freshwater fishes. *Zoological Journal of the Linnean Society, 188*, 345–411. 10.1093/zoolinnean/zlz0039

[CR28] Lebedeva, D., Muñoz, G., & Lumme, J. (2021). New salinity tolerant species of *Gyrodactylus* (Platyhelminthes, Monogenea) on intertidal and supratidal fish species from the Chilean Coast. *Acta Parasitologica, 66*, 1021–1030. 10.1007/s11686-021-00347-x 33792830 10.1007/s11686-021-00347-x

[CR29] Lebedeva, D., Ziętara, M., Mendsaikhan, B., Erlomenko, A., & Lumme, J. (2023). Survivors from a Pliocene climatic catastrophe: *Gyrodactylus* (Platyhelminthes, Monogenea) parasites of the relict fishes in the central Asian internal drainage basin in Mongolia. *Diversity, 15*(7), 860. 10.3390/d15070860

[CR30] Maduenyane, M., Dos Santos, Q. M., & Avenant-Oldewage, A. (2023). *Gyrodactylus sprostonae* Ling, 1962 infect an indigenous cyprinid in southern Africa. An expanded description. *Journal of Helminthology, 97*, e40. 10.1017/S0022149X23000202 10.1017/S0022149X23000202 37199513

[CR31] Matějusová, I., Gelnar, M., McBeath, A. J., Collins, C. M., & Cunningham, C. O. (2001). Molecular markers for gyrodactylids (Gyrodactylidae: Monogenea) from five fish families (Teleostei). *International Journal for Parasitology, 31*, 738–745. 10.1016/s0020-7519(01)00176-x 11336756 10.1016/s0020-7519(01)00176-x

[CR32] Nguyen, L. T., Schmidt, H. A., Von Haeseler, A. & Minh, B. Q. (2015). IQ-TREE: A fast and effective stochastic algorithm for estimating maximum-likelihood phylogenies. *Molecular Biology and Evolution, 32*, 268–274. 10.1093/molbev/msu300 25371430 10.1093/molbev/msu300 PMC4271533

[CR33] Paladini, G., Huyse, T., & Shinn, A. (2011). *Gyrodactylus salinae* n. sp. (Platyhelminthes: Monogenea) infecting the south European toothcarp *Aphanius fasciatus* (Valenciennes) (Teleostei, Cyprinodontidae) from a hypersaline environment in Italy. *Parasites & Vectors, 4*, 100. 10.1186/1756-3305-4-100 10.1186/1756-3305-4-100 PMC3135561 21658217

[CR34] Pleijel, F., Jondelius, U., Norlinder, E., Nygren, A., Oxelman, B., Schander, C., Sundberg, P., & Thollesson, M. (2008). Phylogenies without roots? A plea for the use of vouchers in molecular phylogenetic studies. *Molecular Phylogenetics and Evolution, 48*, 369–371. 10.1016/j.ympev.2008.03.024 18424089 10.1016/j.ympev.2008.03.024

[CR35] Přikrylová, I., Blažek, R., & Vanhove, M. (2012b). An overview of the *Gyrodactylus* (Monogenea: Gyrodactylidae) species parasitizing African catfishes, and their morphological and molecular diversity. *Parasitology Research, 100*(3), 1185–1200. 10.1007/s00436-011-2612-0 10.1007/s00436-011-2612-0 21850451

[CR36] Přikrylová, I., Smit, N. J., & Gelnar, M. (2017). Description of *Afrogyrodactylus ardae* sp. n. (Monogenea: Gyrodactylidae) from *Rhabdalestes septentrionalis* (Characiformes: Alestidae) in the Niokolo-Koba National Park, Senegal. *Helminthologia, 54*, 330–335. 10.1515/helm-2017-0045

[CR37] Přikrylová, I., Matějusová, I., Jarkovský, J. & Gelnar, M. (2008). Morphometric comparison of three members of the *Gyrodactylus nemachili*-like species groug (Monogenea: Gyordactylidae) on *Barbatula barbatula* L. in the Czech Republic, with a reinstatement of *G. papernai* Ergens & Bychowsky, 1967. *Systematic Parasitology, 69*(1), 33–44. 10.1007/s11230-007-9106-6 10.1007/s11230-007-9106-6 18030600

[CR38] Přikrylová, I., Blažek, R., & Gelnar, M. (2012a). *Gyrodactylus malalai* sp. nov. (Monogenea: Gyrodactylidae) from Nile tilapia, *Oreochromis niloticus* (L.) and Redbelly tilapia, *Tilapia zillii* (Gervais) (Teleostei: Cichlidae) in the Lake Turkana, Kenya*. Acta Parasitologica, 57*(2), 122–130. 10.2478/s11686-012-0017-6 10.2478/s11686-012-0017-6 22807048

[CR39] Přikrylová, I., Vanhove, M., Janssens, S. B., Billeter, P., & Huyse, T. (2013). Tiny worms from a mighty continent: High diversity and new phylogenetic lineages of African monogeneans. *Molecular Phylogenetics and Evolution, 67*, 43–52. 10.1016/j.ympev.2012.12.017 10.1016/j.ympev.2012.12.017 23287552

[CR40] Prudhoe, S., & Hussey, C. G. (1977). Some parasitic worms in freshwater fishes and fish-predators from the Transvaal, South Africa. *Zoologica Africana, 12*, 113–147.

[CR41] Rahmouni, S., Seifertová, M., Benovics, M., & Šimková, A. (2023). Diversity and Phylogeny of *Gyrodactylus* spp. (Monogenea: Gyrodactylidae) across the Strait of Gibraltar: Parasite speciation and historical biogeography of West Mediterranean cyprinid hosts. *Diversity, 15*, 1152. 10.3390/d15111152

[CR42] Raphahlelo, M. E., Přikrylová, I., & Matla, M. M. (2020). *Dactylogyrus* spp. (Monogenea, Dactylogyridae) from the gills of *Enteromius* spp. (Cypriniformes, Cyprinidae) from the Limpopo Province, South Africa with descriptions of three new species. *Acta Parasitologica, 65*, 396–412. 10.2478/s11686-020-00175-5 32056086 10.2478/s11686-020-00175-5

[CR43] Řehulková, E., Seifertová, M., Přikrylová, I., & Francová, K. (2018). Monogenea. In: A guide to the parasites of African freshwater fishes. Scholz, T., Vanhove, M. P. M., Smit, N., Jayasundera, Z., & Gelnar, M. (Eds.), *RBINS Scientific Publication Unit* (pp. 185–243). ABC Taxa: Brussels..

[CR44] Ren, Q., Mayden, R. L. (2016). Molecular phylogeny and biogeography of African diploid barbs, ‘*Barbus*’, and allies in Africa and Asia (Teleostei: Cypriniformes). *Zoologica Scripta*, 45, 642–649. 10.1111/zsc.12177

[CR45] Rodriguez, F., Oliver, J. L., Marin, A., & Medina, J. R. (1990). The general stochastic model of nucleotide substitution. *Journal of Theoretical Biology, 142*, 485–501. 10.1016/s0022-5193(05)80104-3 2338834 10.1016/s0022-5193(05)80104-3

[CR46] Rokicka, M., Lumme, J., & Zietara, M. S. (2007). Identification of *Gyrodactylus* ectoparasites in Polish salmonid farms by PCR-RFLP of the nuclear ITS segment of ribosomal DNA (Monogenea, Gyrodactylidae). *Acta Parasitologica, 52*, 185–195. 10.2478/s11686-007-0032-1

[CR47] Ronquist, F., & Huelsenbeck, J. P., (2003). MrBayes 3: Bayesian phylogenetic inference under mixed models. *Bioinformatics, 19*, 1572–1574. 10.1093/bioinformatics/btg180 12912839 10.1093/bioinformatics/btg180

[CR48] Dos Santos, Q. M., Maina, J. N., & Avenant-Oldewage, A. (2019). *Gyrodactylus magadiensis* n. sp. (Monogenea, Gyrodactylidae) parasitizing the gills of *Alcolapia grahami* (Perciformes, Cichlidae), a fish inhabiting the extreme environment of Lake Magadi, Kenya. *Parasite, 26*, 76. 10.1051/parasite/2019077 10.1051/parasite/2019077 PMC6924288 31859621

[CR49] Schedel, F. D. B., Musilová, Z., Indermaur, A., Bitja-Nyom, A. R., Salzburger, W., Schliewen, U. K. (2022). Towards the phylogenetic placement of the enigmatic African genus *Prolabeops* Schultz, 1941. *Journal of Fish Biology, 101,* 1333–1342. 10.1111/jfb.15205 36053860 10.1111/jfb.15205 PMC9826184

[CR50] Shigoley, M. I., Rahmouni, I., Louizi, H., Pariselle, A., & Vanhove, M. P. M. (2023). First study on *Gyrodactylus* (Monogenea: Gyrodactylidae) in Morocco, with description of a new species from *Luciobarbus pallaryi* and *Luciobarbus ksibi* (Actinopterygii: Cyprinidae). *Animals, 13*, 1624. 10.3390/ani13101624 37238053 10.3390/ani13101624 PMC10215732

[CR51] Skelton, P. H., Swartz, E. R., Vreven, E. J. (2018). The identity of *Barbus capensis* Smith, 1841 and the generic status of southern African tetraploid cyprinids (Teleostei, Cyprinidae). *European Journal of Taxonomy, 410*, 1–29. 10.5852/ejt.2018.410

[CR52] Skelton, P. H. (2001). *A complete guide to the freshwater fishes of southern Africa*. 2nd Edition, Struik, Cape Town.

[CR53] Tavaré, S. (1986). Some probabilistic and statistical problems in the analysis of DNA sequences. In: Miura, R.M. (Ed.), *Some mathematical questions in biology—DNA sequence analysis* (pp. 57–86). American Mathematical Society, Providence, Rhode Island.

[CR54] Truter, M., Přikrylová, I., Malherbe, W., & Smit, N. J. (2016). First report of metazoan parasites from the cichlid *Pseudocrenilabrus philander* and cyprinid *Enteromius paludinosus* in a South African Ramsar wetland. *African Journal of Aquatic Sciences, 41*, 499–503. 10.2989/16085914.2016.1246357

[CR55] Truter, M., Smit, N. J., Malherbe, W., & Přikrylová, I. (2022). Description of *Gyrodactylus paludinosus* sp. nov. (Monogenea: Gyrodactylidae) from the Straightfin Barb, *Enteromius paludinosus* (Peters, 1852), in South Africa. *Acta Parasitologica*, *67*, 446–453. 10.1007/s11686-021-00480-7 34677799 10.1007/s11686-021-00480-7

[CR56] Truter, M., Přikrylová, I., Hatfield, K. A., & Smit, J. (2023). Working towards a conservation plan for fish parasites: Cyprinid parasites from the South African Cape Fold freshwater ecoregion as a case study. *International Journal for Parasitology: Parasites and Wildlife, 21*, 277–286. 10.1016/j.ijppaw.2023.07.003 37533698 10.1016/j.ijppaw.2023.07.003 PMC10393515

[CR57] Vaughan, D. B., Christison, K. W., Hansen, H., & Shinn, A. P. (2010). *Gyrodactylus eyipayipi* sp. n. (Monogenea: Gyrodactylidae) from *Syngnathus acus* (Syngnathidae) from South Africa. *Folia Parasitologica, 57*, 11–15. 10.1441/fp.2010.002 20449995 10.14411/fp.2010.002

[CR58] Yang, L., Sado, T., Vincent Hirt, M., Pasco-Viel, E., Arunachalam, M., Li, J., Wang, X., Freyhof, J., Saitoh, K., Simons, A. M., Miya, M., He, S., Mayden, R. L. (2015). Phylogeny and polyploidy: resolving the classification of cyprinine fishes (Teleostei: Cypriniformes). *Molecular Phylogenetics and Evolution, 85*, 97–116. 10.1016/j.ympev.2015.01.014 25698355 10.1016/j.ympev.2015.01.014

[CR59] Zahradníčková, P., Barson, M., Luus-Powell, W. J., & Přikrylová, I. (2016). Species of *Gyrodactylus* von Nordmann, 1832 (Platyhelminthes: Monogenea) from cichlids in Zambezi and Limpopo river basins from Zimbabwe and South Africa: evidence for unexplored species richness. *Systematic Parasitology, 93*, 679–700. 10.1007/s11230-016-9652-x 27522367 10.1007/s11230-016-9652-x

[CR60] Zhang, W., Hao, C., Arken, K., Rong, M., Tian, S., Kadir, M., & Yue, C. (2023). New species of *Gyrodactylus* von Nordmann, 1832 (Monogenoidea: Gyrodactylidae) from *Gymnodiptychus dybowskii* (Kessler, 1874) (Schizothoracinae) in the Kunes River (Yili River basin), China. *International Journal for Parasitology: Parasites and Wildlife, 22,* 136–145. 10.1016/j.ijppaw.202.10.002 37869061 10.1016/j.ijppaw.2023.10.002 PMC10587675

[CR61] Ziętara, M., Kuusela, J., Veselov, A., & Lumme, J. (2008). Molecular faunistics of accidental infections of Gyrodactylus Nordmann, 1832 (Monogenea) parasitic on salmon *Salmo salar* L. and brown trout *Salmo trutta* L. in NW Russia. *Systematic Parasitology, 69*(2), 123–135. 10.1007/s11230-007-9121-7 10.1007/s11230-007-9121-7 18038199

[CR62] Ziętara, M., & Lumme, J. (2002). Speciation by host switch and adaptive radiation in a fish parasite genus *Gyrodactylus* (Monogenea, Gyrodactylidae). *Evolution, 56*(12), 2445–2458. 10.1111/j.0014-3820.2002.tb00170.x 12583585 10.1111/j.0014-3820.2002.tb00170.x

